# Magnetic Resonance Imaging in Primary Progressive Multiple Sclerosis Patients: Review

**DOI:** 10.1007/s00062-022-01144-3

**Published:** 2022-03-08

**Authors:** Malgorzata Siger

**Affiliations:** Department of Neurology, Medical University of Łódź, 22 Kopcinskiego Str., 90-153 Łódź, Poland

**Keywords:** Diagnosis of multiple sclerosis, Differential diagnosis

## Abstract

The recently developed effective treatment of primary progressive multiple sclerosis (PPMS) requires the accurate diagnosis of patients with this type of disease. Currently, the diagnosis of PPMS is based on the 2017 McDonald criteria, although the contribution of magnetic resonance imaging (MRI) to this process is fundamental. PPMS, one of the clinical types of MS, represents 10%–15% of all MS patients. Compared to relapsing–remitting MS (RRMS), PPMS differs in terms of pathology, clinical presentation and MRI features. Regarding conventional MRI, focal lesions on T2-weighted images and acute inflammatory lesions with contrast enhancement are less common in PPMS than in RRMS. On the other hand, MRI features of chronic inflammation, such as slowly evolving/expanding lesions (SELs) and leptomeningeal enhancement (LME), and brain and spinal cord atrophy are more common MRI characteristics in PPMS than RRMS. Nonconventional MRI also shows differences in subtle white and grey matter damage between PPMS and other clinical types of disease. In this review, we present separate diagnostic criteria, conventional and nonconventional MRI specificity for PPMS, which may support and simplify the diagnosis of this type of MS in daily clinical practice.

## Introduction

In recent years, there has been a breakthrough in the treatment of primary progressive multiple sclerosis (PPMS). Ocrelizumab, a humanized monoclonal antibody that selectively depletes CD-20-expressing B cells, reduces the relapse rate and magnetic resonance activity compared to placebo [1]. Additionally, long-term follow-up observations from the study showed that early introduced therapy delayed the time to confirmed disability progression by 5–6 years [2]. The effectiveness of another anti-CD20 antibody, rituximab (RTX), was also evaluated in PPMS patients. Although this study did not show a significant effect on disability progression, in the PPMS group as a whole a subanalysis of younger patients (aged ≤ 51 years) with magnetic resonance activity indicated a possible positive effect [3]. However, to optimize the treatment of patients with PPMS, appropriate diagnostic work-up and effective monitoring of the treatment are necessary.

Magnetic resonance imaging (MRI) is an established paraclinical tool in MS diagnosis and treatment monitoring [4, 5]. The diagnosis and differential diagnostics of PPMS are different from those for relapsing-remitting multiple sclerosis (RRMS) [4, 6]; however, some characteristic features in brain and spinal cord MRI in PPMS have been observed [7, 8].

This review presents the most characteristic features of conventional and nonconventional brain and spinal cord MRIs observed in PPMS patients.

## Search Strategy and Selection Criteria

English language articles published between 1 January 1980 and 31 June 2021 were selected from PubMed (https://www.ncbi.nlm.nih.gov/pubmed), and the reference lists were searched for relevant articles. The search terms “primary progressive multiple sclerosis” [ALL Fields] AND “magnetic resonance imaging” [ALL Fields] were used. Reviews and original articles were included. Case studies were excluded. The final reference list was based on relevance to the theme of this review.

## Diagnosis and Classification of PPMS

On the basis of the last accepted diagnostic criteria (McDonald revision 2017) [4], PPMS can be recognized if clinical progression of the disease has been observed for at least 1 year and 2 of 3 additional criteria have been met (Table 1).

**Table 1:** The 2017 McDonald diagnostic criteria for multiple sclerosis [4]

**Relapsing-remitting multiple sclerosis**		
	*Number of lesions with objective clinical evidence*	*Additional data needed for a diagnosis of multiple sclerosis*
≥ 2 clinical attacks	≥ 2	None
≥ 2 clinical attacks	1 (as well as clear-cut historical evidence of a previous attack involving a lesion in a distinct anatomical location)	None
≥ 2 clinical attacks	1	Dissemination in space demonstrated by an additional clinical attack implicating a different CNS site or by MRI^a^
1 clinical attack	≥ 2	Dissemination in time demonstrated by an additional clinical attack or by MRI^b^ OR demonstration of CSF-specific oligoclonal bands
1 clinical attack	1	Dissemination in space demonstrated by an additional clinical attack implicating a different CNS site or by MRI^a^ AND Dissemination in time demonstrated by an additional clinical attack or by MRI^b^ OR demonstration of CSF-specific oligoclonal bands
**Primary progressive multiple sclerosis**		
Clinical presentation	≥ 1 year of disease progression, which can be determined either prospectively or retrospectively with 2 of the 3 following criteria:	
MRI criteria	1. One or more T2-hyperintense lesions characteristic of multiple sclerosis in one or more typical locations^c^ 2. Dissemination in time on spinal cord MRI (at least two T2-hyperintense lesions) 3. Positive results from the cerebrospinal fluid analysis (i.e., presence of oligoclonal bands)	

^a^Dissemination in space (DIS): one or more T2-hyperintense lesions that are characteristic of multiple sclerosis in two or more of four areas of the CNS: 1. periventricular, 2. cortical or juxtacortical, 3. infratentorial, 4. spinal cord

^b^Dissemination in time (DIT): the simultaneous presence of gadolinium-enhancing and nonenhancing lesions at any time or by a new T2-hyperintense or gadolinium-enhancing lesion on follow-up MRI, with reference to a baseline scan, irrespective of the timing of the baseline MRI

^c^1. cortical or juxtacortical, 2. periventricular, 3. infratentorial

It is worth stressing that to meet dissemination in space (DIS) criteria for PPMS, a lower number of brain focal lesions is required than for RRMS [4]. Other differences between RRMS and PPMS diagnostic criteria are presented in Table 2. Additionally, unlike the 2010 McDonald criteria [6] no distinction between symptomatic and asymptomatic lesions is necessary [4].

**Table 2:** Differences between RRMS and PPMS diagnostic criteria

	RRMS	PPMS
*Clinical presentation*	At least 1 clinical attack	1 year of disease progression, clinical attack is not obligatory
*Results from additional examination*		
Brain MRI (DIS)	≥ 2 lesion (required)	≥ 1 lesion (not required)
Spinal cord MRI (DIS)	≥ 1 lesion, possible	≥ 2 lesions, one of the paraclinical criteria

*RRMS* relapsing remitting multiple sclerosis,* PPMS *primary progressive multiple sclerosis, *MRI *magnetic resonance imaging, *DIS *dissemination in space

In 2013, Lublin et al. proposed a new classification for the clinical form of MS [9]. The PPMS and secondary progressive MS (SPMS) were included as one MS phenotype, i.e., progressive multiple sclerosis (PMS). Depending on the evidence of disease progression and the features of disease activity, the following subtypes of PMS were described: not active without progression, not active with progression, active without progression and active with progression, where MS activity was defined as MS relapse and/or features of MS activity on MRI (contrast-enhancing lesions and/or new/enlarging T2 lesions). Disease activity was assessed once a year or more frequently, depending on the course of MS. An MS progression was defined as a documented gradual increase in neurological deficits per expanded disability status scale (EDSS) that persists for 3–6 months.

## MRI Characteristics of PPMS Patients

### Brain

#### Gadolinium-Enhancing Lesions (Gd+ Lesions)

**Fig. 1:**
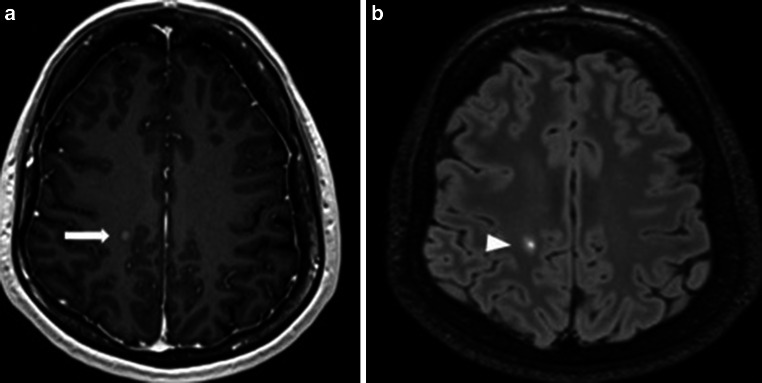
Active MS lesion (Gd+ lesion) in a primary progressive MS patient. **a** Axial T1-weighted image after contrast administration in a PPMS patient shows a gadolinium-enhancing lesion (*white arrow*) in juxtacortical white matter. **b** Corresponding section of axial fluid attenuated inversion recovery (FLAIR) image with hyperintense white matter lesion (*arrowhead*) in the same location

The diagnostic criteria of PPMS are distinct from those of RRMS [4]. The most important arguments for this distinction are based on neuropathological studies that showed that the pathological processes in PPMS seem to differ from those in RRMS and even those in SPMS [10, 11]. These differences can also be seen in MRI [12, 13]. One such differentiating feature is the presence of gadolinium-enhancing lesions (Gd+). These represent areas of active inflammation, which are more characteristic of RRMS patients. In PPMS patients as well as in the early and late phases of disease, the presence of Gd+ lesions is less frequent than in RRMS patients. In a study by Ingle et al. [14] Gd+ lesions were detected in approximately 42% of patients with PPMS; however, the disease duration was no longer than 5 years and a triple dose of contrast agent was administered. In the follow-up of this study, Khaleeli et al. [15] found that the percentage of patients with Gd+ lesions in the brain and spinal cord decreased from 33% to 26% over 5 years. The authors of both studies indicated that in PPMS patients in the early phase of disease there is also active inflammation (expressed as the occurrence of Gd+ lesions) and that this active phase declines during progression of the disease (which is expressed as a reduction in the number of Gd+ lesions). It is worth noting that neurological progression measured by the EDSS was associated with a greater number of Gd+ lesions at baseline [15] but even with techniques that improved the sensitivity of contrast enhancement, Gd+ lesions are often absent despite continuing clinical deterioration [16–18]. Support for a lower number of Gd+ lesions in the late phase of PPMS is derived from treatment trials. In the PROMIse trial, 14% of all baseline MRIs from 943 patients had Gd+ lesions [19]. In the OLYMPUS trial, Gd+ lesions were observed in 24.5% of baseline scans [3]. Finally, in the ORATORIO study, depending on the inclusion/exclusion criteria, Gd+ lesions were found in 24.7–27.5% of patients before treatment initiation [1]. In all studies mentioned above the number of PPMS patients with Gd+ lesions was much less than 75% of RRMS patients who enhanced in early phase of disease [20]. Very recently published analysis of four large randomized datasets showed that higher age of patients was associated with lower risk of Gd+ lesions at baseline [21]. An example of active inflammation expressed by Gd+ lesions in PPMS patients is presented in Fig. 1.

#### Focal T2-weighted Lesions

**Fig. 2:**
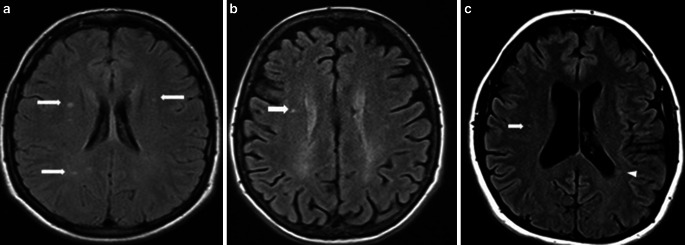
Focal white matter lesions in three PPMS patients. **a** Axial fluid attenuated inversion recovery (FLAIR) images with only a few small, focal white matter lesions located in deep white matter in right and left brain hemisphere (*white arrows*), **b** axial FLAIR image with punctate lesion in right deep white matter (*white arrow*), **c** axial FLAIR image with small, focal lesion located in deep white matter of right brain hemisphere (*white arrow*) and in periventricular white matter (*white arrowhead*) of left brain hemisphere

**Fig. 3:**
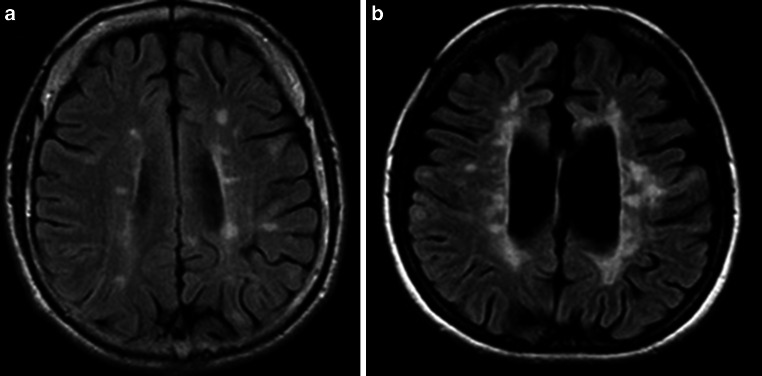
Brain MRI scans in PPMS patients who are similar to those seen in relapse–remitting multiple sclerosis (RRMS) and secondary progressive multiple sclerosis (SPMS). Brain MRI scans of PPMS patients with different MRI presentations **a** FLAIR image, axial plane. Multiple focal lesions were located in the periventricular, deep and juxtacortical white matter. This MRI is similar to a typical MRI scan observed with RRMS. **b** Fluid attenuated inversion recovery (FLAIR) image, axial plane. Focal and confluent white matter lesions were located in the periventricular white matter; some lesions located in the juxtacortical regions were similar to those seen in SPMS. Signs of brain atrophy are also visible

The most common feature in conventional MRI in PPMS patients is a low number of focal brain lesions on T2-weighted images ([22]; Fig. 2); however, there were also some MRIs of PPMS patients with a high number of focal brain lesions that resembled the images of RRMS or even SPMS patients (Fig. 3). Lesions are located in different parts of the brain but in many PPMS patients they are found in the regions responsible for motor functions [23].

**Fig. 4:**
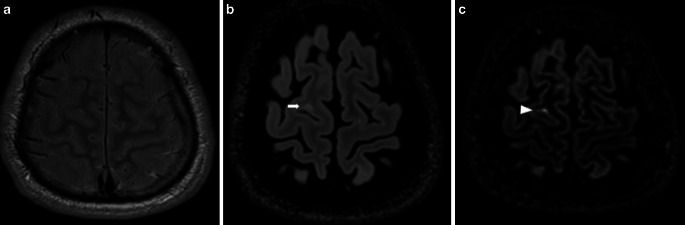
Cortical/juxtacortical lesions in a patient with PPMS. MRI scan in a patient with PPMS. **a** Proton-density (PD)-weighted image in the axial plane. Nonvisible cortical lesion in the right parietal region. **b** Fluid attenuated inversion recovery (FLAIR) image, axial plane. A poorly visible cortical lesion in the right parietal region (*white arrow*). **c** Double inversion recovery (DIR) image of a clearly visible lesion in the same location (*white arrowhead*)

Similar to what is observed in RRMS and SPMS patients, PPMS patients have focal lesions located in the periventricular and deep white matter [24, 25]; however, in contrast to RRMS and SPMS patients, the characteristic location of the focal brain lesions in PPMS patients is the juxtacortical/cortical areas ([22]; Fig. 4). It has been estimated that over 80% of patients with PPMS have lesions in this location [22]. The occurrence of cortical lesions has been strongly associated with more advanced neurological deficits and cognitive dysfunctions [23, 26]; however, to detect cortical lesions more sensitive sequences, such as 3D double inversion recovery (DIR) and phase-sensitive inversion recovery (PSIR), are recommended [27, 28].

On the basis of the results from immunochemical studies, cortical lesions are classified into four subtypes [29]: type 1: leukocortical; type 2: intracortical; type 3: subpial; and type 4: large cortex-spanning lesions. Types 3 and 4 are the most common lesion types found in patients with PMS; however, in vivo detection of these lesions is complicated even using an ultrahigh-field MRI scanner (7T). A study showed that types 3 and 4 were the most frequently detected (50.2%), followed by type 1 (36.2%) and type 2 (13.6%) lesions [30]. The formation of subpial lesions may be independent of white matter lesions and has been proposed as a characteristic MRI feature of the corticospinal variant of MS [23].

#### Leptomeningeal Enhancement

Histological studies in the past several years have shown an association between the presence of cortical subpial lesions and leptomeningeal inflammation (LMI), mainly in patients with progressive MS [31–36]. The detection of LMI in conventional MRI is very difficult. The recommended MRI sequence is high-resolution, postcontrast T2 FLAIR with a 10-min delay after gadolinium administration [37–39]. Using 3D postcontrast T2 FLAIR imaging, it is possible to detect more LME in MS patients compared to conventional T1-weighted imaging [37]. In a very elegant and interesting study, Absinta et al. [37] detected LME in 24.7% of patients with MS and in only 2.7% of age-matched controls without MS. A very important finding from this study was that LME was more characteristic for patients with progressive MS than in RRMS patients (33% vs. 19%, respectively). Moreover, the highest prevalence was observed in PPMS patients (38% of patients). They also showed that certain clinical parameters, such as neurological disability, disease duration and higher age were associated with the presence of LME. Additionally, in patients with LME brain volume loss and atrophy in the cerebral cortex were detected but there was no association between the presence of LME and white matter lesion volume and enhancement. From a clinical perspective, a very important finding comes from the follow-up of this study performed after a mean interval of 1.4 years. The authors detected continuation of enhancement in 85% of LME foci despite disease-modified treatment applied in MS patients.

In work by Zivadinow et al. [39] the presence of LME was evaluated in RRMS and SPMS patients. This study detected LME in half of the patients of whom most (56.0%) had SPMS. Patients with LME had lower global cerebral grey matter volume and cortical grey matter volume in the previous 5 years compared to patients without LME. The presence of LME was not associated with the relapse rate or use of disease-modified therapy. In routine clinical practice, LME is visible on postcontrast 3D FLAIR images obtained on a 3 T scanner; however, using an ultrahigh-field MRI scanner (7T), LME was visualized much better and was seen in up to 90% of MS patients [40]. It is also worth noting that LME is present in other neurological diseases, such as vasculitis [41], tumors [42] and ischemia [43] but the results from both pathological and imaging studies in MS patients indicate that LME represents one form of grey matter characteristic of progressive MS [44, 45].

#### T1-weighted Lesions (“Black Holes”)

**Fig. 5:**
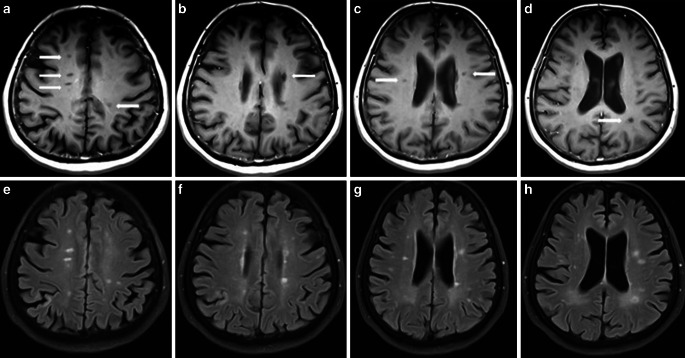
Black holes in four PPMS patients. **a**–**d** Axial T1-weighted spin-echo images with hypointense lesions (black holes) in deep and periventricular white matter (*white arrows*), **e**–**h**. Axial fluid attenuated inversion recovery (FLAIR) images with corresponding hyperintense lesions in the same locations

T1-weighted lesions are defined as areas of hypointense signal intensity on T1-weighted images compared with normally appearing white and grey matter and with corresponding hyperintense white matter areas on T2-weighted images [5, 13]. There are two subclasses of black holes: acute, which reflect transient edema and inflammation and chronic, which are characterized by axonal loss, severe demyelination and matrix destruction [46, 47]. Chronic black holes correlate better with clinical disability [46, 48]. The value of black holes, especially the association with disability and clinical progression, was evaluated mostly in RRMS patients [47, 49–56]. In SPMS patients, an increase in the lesion volume of black holes was identified as a marker of progression from relapsing-remitting to secondary progression MS [13]. In PPMS, new lesions showing hypointensity on T1-weighted MRI scans after 15 months were associated with neurological worsening measured by the EDSS score after 15 years [51]. An example of black holes in PPMS patients is presented in Fig. 5.

#### “Dirty Appearing White Matter” (DAWM)

**Fig. 6:**
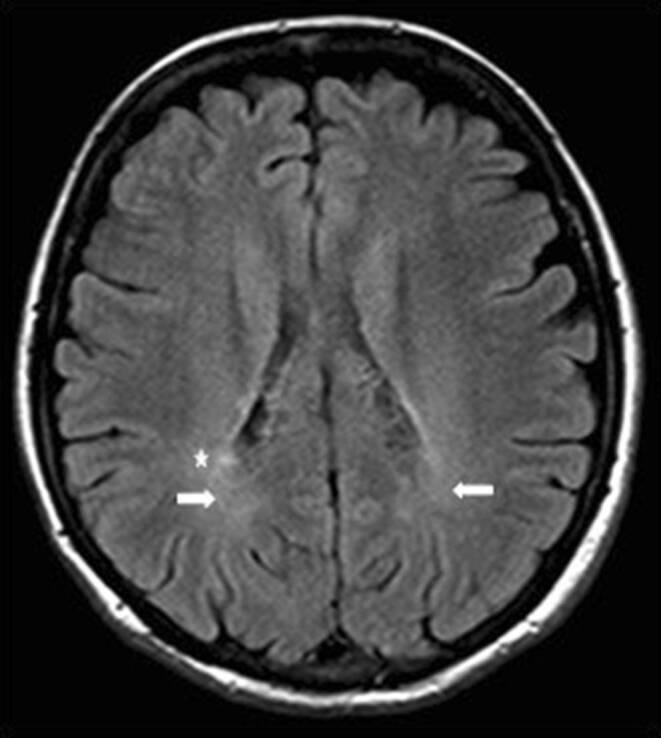
Dirty appearing white matter (DAWM) in a patient with PPMS. Fluid attenuated inversion recovery (FLAIR) image in the axial plane. Around the posterior horns of the lateral ventricles, ill-defined areas of increased signal intensity are visible (*white arrow*). Right periventricular hyperintense focal lesions are also visible (*asterisk*)

Other characteristic MRI features in PPMS patients are ill-defined areas of increased signal intensity in T2-weighted or proton density-weighted images located primarily in the region of the lateral ventricles, especially in the parieto-occipital region or in the centrum semiovale [57–59]. These signal abnormalities are termed “dirty appearing white matter” (DAWM) [13]. A histopathologic analysis of DAWM showed a broad spectrum of abnormalities, including inflammation, demyelination, blood–brain barrier disruption, gliosis, and axonal loss [60]; however, these processes were less severe than those observed in focal white matter lesions [57]. It was also demonstrated that DAWM was secondary to neuronal and axonal damage in focal white and grey matter lesions [61]. An example of DAWM in a PPMS patient is shown in Fig. 6.

#### Slowly Expanding/Evolving Lesions (SELs)

Neuropathological evidence has indicated that chronic inflammation is a pathological natural continuum of active inflammation and a process that is more prominent in the progressive form of MS [61, 62]. Conventional MRI, even with gadolinium contrast agent injection, is relatively insensitive to chronic inflammation [62]. In light of the apparently diminished inflammatory nature of PPMS, quantitative measurements using fast serial scans to detect blood–brain barrier disruption in the location of chronic inflammation were performed [63]; however, these serial contrast agent measurements are difficult to utilize in clinical practice. Another quantitative MRI measurement based on the assessment of longitudinal changes in lesion size has shown promising results. Slowly expanding/evolving lesions (SELs) are not enhanced after contrast agent administration [63]. The SELs are prominent in progressive MS patients (in 12–28% of plaques), especially in patients with a disease duration of more than 10 years who are over 50 years of age [63]. Histological studies have shown that SELs are characterized by an inactive center with few or no macrophages surrounded by a rim of activated microglia/macrophages that slowly expand over time [64]. There is no consensus regarding the in vivo detection of SELs. In T1-weighted images, SELs tend to have a lower signal intensity than nonexpanding lesions. One of the options for depicting these lesions is susceptibility-weighted imaging (SWI) [65]. The SELs are best detected using a 7T scanner but can also be identified with a 3T magnet [66]. Recently, Eliot et al. described a very promising method to detect and quantify SELs based on T1-weighted and T2-weighted images [67]. On the basis of this new method, the authors showed that PPMS patients had a higher mean number of SELs than RRMS patients (6.3 vs. 4.6, *p* = 0.02), a higher T2 volume of SELs (baseline: 1838 mm^3^ vs. 1223 mm^3^, *p* < 0.001), and a higher mean proportion of baseline total T2 lesion burden detected as SELs (11.3% vs. 8.6%, *p* < 0.001). The SELs evolved independently from Gd+ lesions with a progressive decrease in T1 intensity over time. The accumulation of SELs differs across the brain. In PPMS patients, SELs are mainly localized in the periventricular posterior part of the brain [67]. A characteristic MRI feature of chronic inflammation is white matter lesions, hyperintense on T2-weighted images with hypointense peripheral rims, termed as rim-positive or rim-like lesions. The characteristic feature of these lesions is a hypointense, paramagnetic rim, which is related to iron deposition in protein and myelin [68]. The basic MRI sequence to detect rim-positive lesions is high-resolution T2*/SWI. The optional methods are SWI sequences with quantitative measures such as quantitative susceptibility mapping (QSM) and R2* (transverse relaxivity-inverse of T2*) [68]. These rim-like or rim-positive lesions were first detected with T2* and SWI sequences [69, 70]. An analysis of MS lesions based on SWI showed that 10–20% of lesions were rim-positive [70, 71]. Rim-positive lesions were detected in all types of MS but some studies have reported higher rates of rim-like lesions in PMS patients than in RRMS patients [68].

The presence of rim-like lesions has clinical implications. In a study by Absinta et al. [72], the authors showed that having ≥ 4 rim-positive lesions was associated with motor and cognitive progression in younger patients [72] and that rim-positive patients had a 3.2-fold higher prevalence of PMS than patients with three or fewer rim-like lesions [72]. Additionally, a very recently published study showed that the occurrence of rim-positive lesions in periventricular and subtentorial locations was associated with a higher annual relapse rate [73]. The presence of rim-positive lesions also correlated with higher T2 and T1 lesion volumes and brain atrophy [74].

**Fig. 7:**
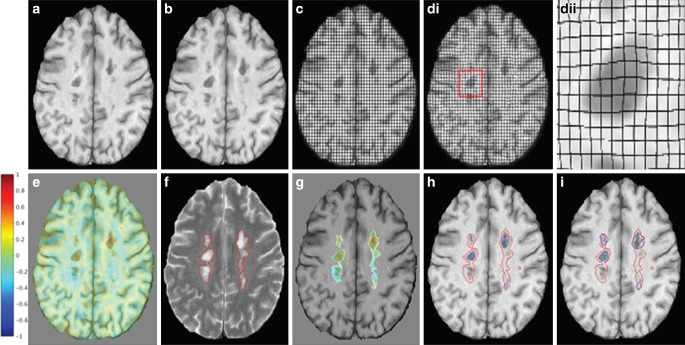
Jacobian analysis and SEL candidates [63] (Reprinted by permission of SAGE Publication). **a**, **b** An axial slice of linearly coregistered reference and follow-up T1-weighted scans. **c** The reference scan with a regular grid overlaid. **di** The nonlinearly deformed image in **c** is shown to match the follow-up scan, and **dii** an enlarged lesion area of the deformation field. **e** The Jacobian determinant is shown as a heat map, where blue represents local contraction and red represents local expansion. The Jacobian determinant represents the local percent volume change at each voxel after application of the nonlinear deformation that warps (**a**) to match (**b**). **f** An axial slice of a reference T2-weighted scan with overlaid T2 lesion segmentation. **g** The Jacobian determinant within reference T2 lesions. **h** Initial SEL candidate boundaries based on JE1. **i** Refined SEL candidate boundaries based on JE2. *JE* Jacobian expansion, *SEL* slowly expanding/evolving lesion

An example of SELs in a PPMS patient is presented in Fig. 7**.**

#### Brain Atrophy

Brain atrophy represents irreversible neurodegenerative processes measured in vivo using MRI as a reduction in brain volume [75, 76]. Brain volume loss in MS patients depends on clinical phenotypes [77]. In patients with PPMS, the rate of brain atrophy is faster than that in RRMS patients (−0.63–0.94%/year vs. −0.22–1.34%/year) [77, 78]. Additionally, the dynamics of brain atrophy were greater in PPMS patients with more severe neurological deficits and features of disease activity in MRI than in clinically stable patients without MRI features and clinical activity (0.45% vs. 0.38% per year, respectively) [78]. Brain atrophy is not a uniform process. Recent studies have suggested that atrophy in the cerebral cortex is more advanced in PPMS patients than in RRMS patients, which is probably associated with a more rapid progression of disability in PPMS patients [79–81]. Additionally, cortical atrophy in PMS patients proceeded 1.8 times faster than that in RRMS patients [82]. In addition to cortical grey matter, atrophy in PPMS has been detected in deep grey matter structures, such as the thalamus, putamen, hippocampus and cingulate gyrus [83]. Thalamic atrophy occurred in the early phases of the disease and was considered a predictive factor for the development of disability within several years [84, 85]. Similar observations apply to atrophy of the cingulate gyrus. Cortical atrophy of the cingulate gyrus occurs early in the course of the disease and is associated with patient neurological outcomes and cognitive impairment [79, 86]. The role of cerebellar atrophy as a good marker of neurodegeneration in patients with PPM has also been emphasized [87, 88]. In PPMS patients, cerebellar atrophy was significantly correlated with the progression of physical disability and cognitive impairment [87, 88]. This correlation is why some authors have suggested that cerebellar volume measurements, especially cerebellar cortex volume, should become MRI markers for treatment efficacy in patients with PPMS [87]. Examples of whole brain atrophy in PPMS patients are shown in Fig. 3b.

### Spinal Cord MRI in PPMS Patients

#### Focal T2-weighted Lesions

**Fig. 8:**
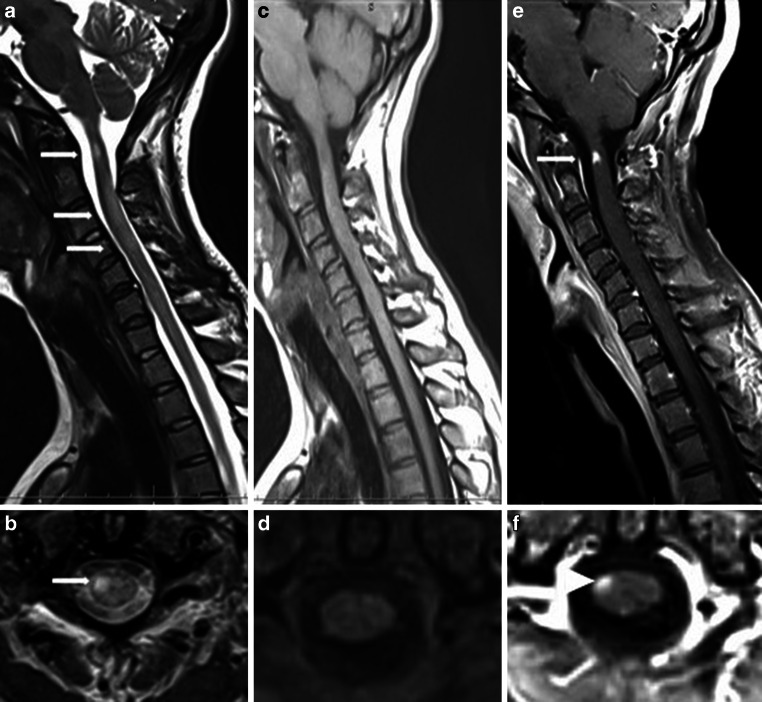
MRI of focal lesions in the spinal cord of a patient with PPMS. **a** T2-weighted, turbo spin-echo image, sagittal plane. Hyperintense focal lesions at the C1 C3, C4, C5 spinal cord levels (*white arrows*). **b** Corresponding T2-weighted turbo spin-echo image, axial section at C1 spinal cord level. Hyperintense focal lesions at right side of spinal cord. **c** T1-weighted, turbo spin-echo, sagittal plane without signal abnormalities and corresponding axial section at C1 spinal cord level (**d**) also without signal abnormalities. **e** T1-weighted images after contrast administration, sagittal plane. Gd+ lesion at C1 spinal cord level (*white arrow*) and axial section at the same spinal cord level (**f**) with Gd+ lesion (*arrowhead*)

In reference to their size, location, and extent, the focal lesions visible on T2-weighted images or short tau inversion recovery images (STIR) in patients with PPMS are similar to those in patients with RRMS [89–91]. It is estimated that focal spinal cord lesions are present in approximately 50% of PPMS patients [89–91]. It is very important to highlight that although in current and very recently published guidelines for diagnosis and monitoring MS patients [92] the sagittal plane is recommended for lesions detection, using additional axial plane more lesions are detected [93, 94]. Galler et al. [93]. applied axial T2-weighted sequences with full spinal cord. One of the limitations of this study was the lack of sequences after contrast agent administration. In a study by Breckwoldt et al. [94] the authors used sequences before and after contrast agent administration, examined patients using sagittal plane and additionally axial acquisition with long coverage of the entire cervical spinal cord. The authors detected 2.5-fold as many lesions on axial than on sagittal sections and in 17% of patients had lesions in the axial plane but these were not visible on sagittal sections. Additionally, they found Gd+ lesions in six patients on axial but not on sagittal scans. Additional application of axial sequences in detection of spinal cord lesions may have clinical value in diagnosis and monitoring the treatment of multiple sclerosis patients. An example of focal lesions, hyperintense on T2-weighted images are shown in Fig. 8a, b.

#### Gadolinium-enhancing Lesions (Gd+ Lesions)

Inflammatory activity is an interesting finding from spinal cord MRIs in PPMS patients. A study performed by Ingle et al. [14] confirmed low spinal cord inflammatory activity: only 6% of PPMS patients in the early phase of the disease had enhancing spinal cord lesions. This activity is very interesting because spinal cord presentation is one of the most common clinical symptoms in this type of MS and does not seem to be associated with typical inflammation detected by conventional MRI techniques. An example of focal Gd+-enhancing lesions is presented in Fig. 8e, f.

#### Diffuse Spinal Cord Abnormalities

**Fig. 9:**
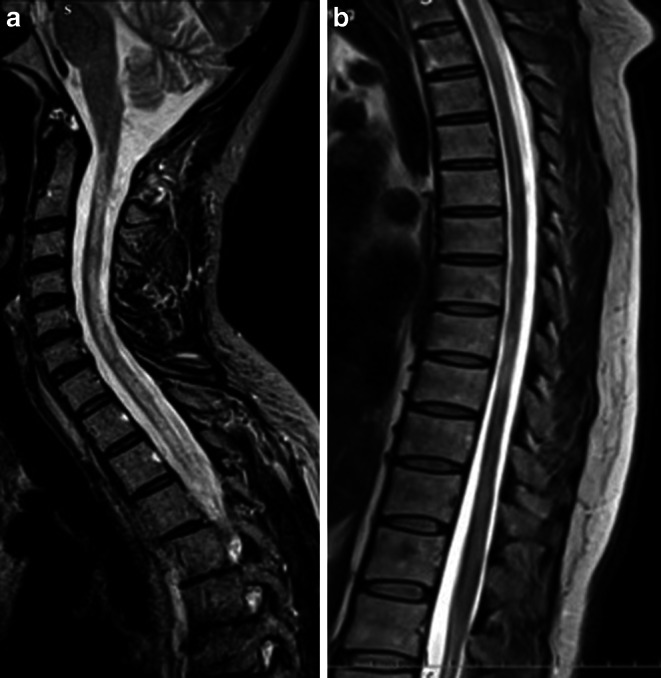
Diffuse abnormalities in the spinal cord of a patient with PPMS. An MRI scan of the spinal cord in a patient with PPMS. **a** Cervical spinal cord, T2-weighted image, sagittal plane. Diffuse, hyperintense signal abnormalities in the whole cervical spinal cord. **b** Thoracic spinal cord; T2-weighted image in the sagittal plane. Characteristic features of diffuse abnormalities with heterogeneous signals at multiple levels and spinal cord atrophy are visible

The unique feature of spinal cord MRI in PPMS patients is diffuse mild hyperintensity in proton-density (PD) or STIR images with a lack of well-demarcated borders [13]. These diffuse spinal cord abnormalities are much more common in spinal cord MRI in PPMS patients than in SPMS and RRMS patients (61% vs. 31% vs. 21%, respectively) [90]. In correlation with the clinical presentation, it was shown that diffuse spinal cord abnormalities were associated with sensorimotor, bowel, and bladder dysfunction [90]. In PPMS patients, a diffuse abnormality in the spinal cord indicates that the pathologic process in the spinal cord is widespread and not restricted to focal lesions [90]. An example of diffuse spinal cord abnormalities is shown in Fig. 9.

#### Spinal Cord Atrophy

**Fig. 10:**
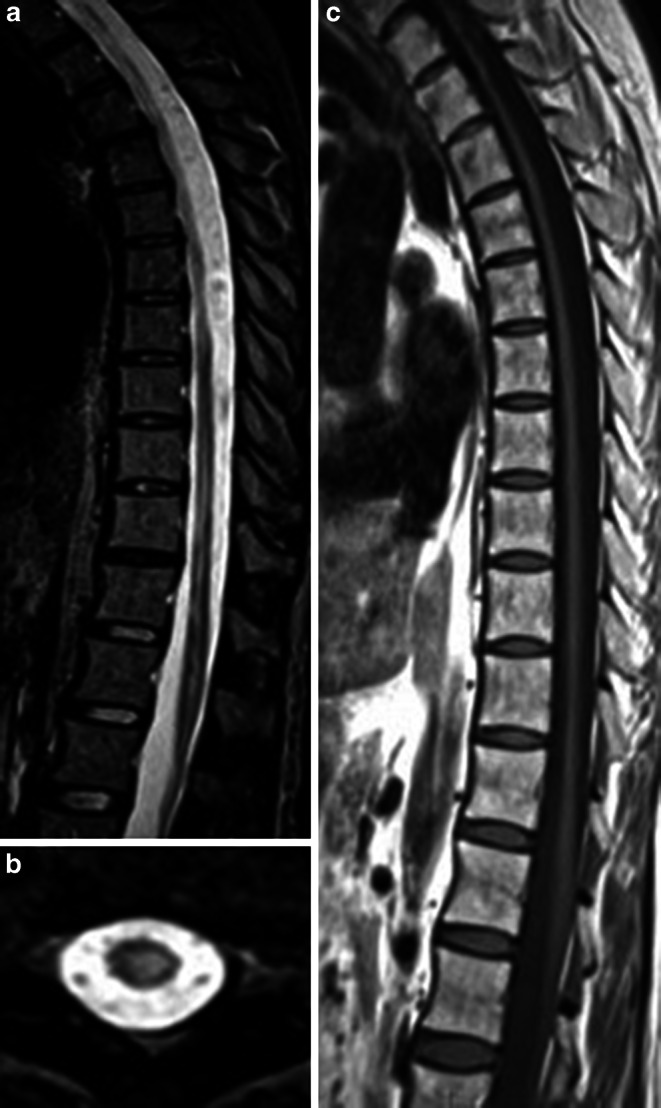
MRI of spinal cord atrophy in a patient with PPMS. Spinal cord atrophy in a patient with PPMS **a** T2-weighted image in the sagittal plane; marked atrophy of the thoracic spinal cord segments with visible hyperintense signal abnormalities. **b** T2-weighted image in the axial plane of thoracic segment (Th9–Th10), marked atrophy with hyperintense signal reflects demyelinating lesion. **c** T1-weighted image, sagittal plane in the same patient with marked atrophy in thoracic spinal cord

The discrepancy between the focal MRI features in relation to the most common original spinal cord MS clinical presentation in PPMS patients may be explained by the occurrence of spinal cord atrophy. It was reported that spinal cord atrophy in PPMS occurs in a very early phase of the disease and progresses more rapidly than in RRMS patients (1–5% vs. 2–3% per year) [89, 95, 96]. It is also important that the progression of spinal cord atrophy, especially spinal cord grey matter in PPMS patients, strongly correlates with an increase in neurological disability [97, 98]. Moreover, Aymerich F et al. [97] in a longitudinal study did not find a correlation between brain and cervical spinal cord atrophy in PPMS patients. These findings may indicate that in PPMS patients, brain and spinal cord pathologies evolve independently and measurements of brain and spinal cord atrophy can provide complementary information about the nature and extent of the disease in PPMS patients [95, 97–99]. An interesting result concerning spinal cord atrophy in different MS clinical phenotypes come from study by Eden et al. [100]. The authors demonstrated the craniocaudal patterns of cervical spinal cord atrophy evolution, starting from the upper cervical cord in RRMS patients and spreading to lower cervical segments in PPMS patients [100]. Moreover, a very recently published study concerning assessment of atrophy along the entire spinal cord in different clinical subgroup of MS patients showed that spinal cord cross-sectional area from C2–C3, C4–C5 and T4–T9 in PPMS patients is significantly lower than in RRMS patients [101]. An example of spinal cord atrophy in a patient with PPMS is shown in Fig. 10.

### Nonconventional MRI (non-cMRI)

Focal brain and spinal cord lesions do not represent the whole pathology of MS [23, 102, 103]. Evidence from both imaging and pathological studies showed that in normal-appearing white matter (NAWM) and normal-appearing grey matter (NAGM), there are subtle abnormalities that are not visible in conventional MRI [104–108]. To explore these changes in vivo, nonconventional MRI techniques, such as magnetization transfer imaging, diffusion-weighted imaging, and proton resonance spectroscopy are often applied [105, 108].

#### Magnetization Transfer Imaging (MTI).

Magnetization transfer imaging is a measure of the magnetization exchange between relatively free water protons and macromolecular protons [105, 109]. The magnitude of this process is expressed as the magnetization transfer ratio (MTR) index. MRI-pathological studies revealed that a decreased MTR value reflects the destruction of myelin and axons [110, 111]. The results from several studies have shown that the MTR in the NAWM and NAGM is lower in patients with MS than in healthy controls [112, 113]. Both MTRs from the NAWM and NAGM were lower in PPMS patients than in RRMS patients, which indicates that axonal pathology is more advanced in the PPMS than in the RRMS form of MS [104, 114]. The MTR in PPMS patients also provided prognostic value. A study performed by Khaleeli et al. demonstrated that a lower mean baseline MTR value from the NAWM and NAGM correlated with a greater deterioration in neurological function as measured by EDSS after 1 year [115, 116]. Additionally, in a study by Rocca et al. [117], a decrease in the MTR value in the grey matter during the first 15 months of observation was a predictor of long-term disability in PPMS patients. Although technically challenging, the MTR has also been used to assess NAWM and NAGM in the spinal cord [104, 106, 118]. The MTR value from the cervical spinal cord in PPMS patients correlated with neurological deficits measured in the EDSS [104, 118]. The MTR histogram parameters were considerably lower in patients with SPMS than in those with PPMS, irrespective of the lack of differences in the size or number of focal lesions visible with conventional MRI sequences [118].

#### Diffusion-Weighted Imaging (DWI).

Diffusion-weighted imaging allows the brain tissue microstructure to be assessed by analyzing its diffusion properties [119]. The degree and orientation of diffusion are measured by two parameters: mean diffusivity (MD), which is a measure of the degree of the restriction to diffusion, and fractional anisotropy (FA), which is a measure of anisotropic diffusion [105, 120].

In accordance with these assumptions, DWI showed increased MD values and decreased FA values within the focal lesions as well as in the NAWM and NAGM in patients with MS [121]. Based on pathological examination, increased MD values and decreased FA values represent the process of demyelination and axonal loss [121, 122]. Multicenter studies in patients with various clinical forms of MS reported that MD values in T2 focal lesions and in the NAWM and NAGM were significantly higher in patients with SPMS than in patients with PPMS [123]. In another study, reduced FA values in the corpus callosum were accompanied by marked cognitive impairment, especially in terms of verbal memory [124]. The DWI metric measurements have also been used as a predictive factor of disability and neurological impairment. In a 5-year follow-up study Rovaris et al. showed that baseline grey matter MD was an independent predictive factor for a rapid increase in neurological deficits [125]. In a study by Rocca et al. using an integrated clinico-imaging model with grey matter MD, new T1-hypointense lesions and percentage brain volume changes predicted long-term disability changes in 77.6% of PPMS patients [126]. Additionally, a gradual decrease in thalamic FA within the first 15 months of disease onset constituted an independent unfavorable predictive factor for disability progression over the next 5 years [127]. Diffusion tensor imaging (DTI) has also been applied to distinguish PPMS from SPMS [128]. This study showed higher interhemispheric differences in MD values in PPMS than in SPMS for certain grey matter structures (e.g., substantia nigra and putamen). As a result of technical limitations, DWI of the spinal cord is not frequently performed. Nevertheless, one study demonstrated lower FA values in the cervical spinal cord and an increase in MD in patients with PPMS compared with patients with RRMS, which indicated differences in spinal cord pathology in these two forms of MS [129].

#### Proton Magnetic Resonance Spectroscopy (H-MRS).

Proton magnetic resonance spectroscopy enables the concentration of selected metabolites to be assessed in various brain regions. The most frequently determined parameters include N‑acetylaspartate (NAA), which is an established marker of axonal damage; choline (Cho)-containing phospholipids, a marker of myelin damage; creatine and phosphocreatine (Cr), indicators of gliosis; myoinositol (mIn), which is a marker of astroglial activation and lactate (LA), a marker of inflammation [130]. Studies in patients with various clinical forms of MS have not yielded unequivocal results. In some cases, there were no differences in metabolite concentrations between patients with different clinical types of MS [131, 132]; however, a study by Suhy et al. demonstrated higher Cho/Cr levels in NAWM in patients with PPMS than in patients with RRMS [133]. Changes in metabolite concentrations, especially in the grey matter of patients with PPMS, were correlated with the degree of neurological deficits [134, 135]. In a study by Rahimian et al. [136] Cr concentrations and the NAA/Cr ratio were significantly lower in chronic nonenhancing focal lesions in patients with PPMS than in patients with RRMS. In another study, decreases in cortical grey matter NAA concentrations were associated with clinical impairments in PPMS patients [134]. In the same study, an increase in myoinositol in NAWM was associated with clinical disability [134]. Using an even more advanced spectroscopic imaging technique, chemical shift imaging, it was demonstrated that the level of glutathione (a major brain antioxidant) was significantly lower in patients with PPMS than in patients with RRMS, which points to a greater role of oxidative stress versus inflammation in the chronic phase of the disease [137].

#### Susceptibility-Weighted Imaging (SWI).

Susceptibility-weighted imaging is another nonconventional MRI technique used to assess patients with PPMS [138, 139]. It enables the indirect assessment of iron levels in selected brain regions. Using SWI sequences, Burgetova A et al. demonstrated that magnetic susceptibility in the thalamus was higher in patients with RRMS than in patients with PPMS, which reflected differences in the thalamic iron concentration between these two forms of the disease [140].

Very recently, another attractive unconventional MRI method for measuring brain damage in PPMS patients was developed. Covariance network analysis, which measures interdependencies across various structures, assesses different aspects of brain damage and may provide information complementary to that from structural MRI methods [141].

A summary of the most characteristic features of brain and spinal cord MRI in PPMS patients is given in Table 3.

**Table 3:** MRI characteristic features of PPMS patients

MRI feature	Characteristic	Reference
**Brain**		
Number of Gd+ lesions	Low	[1–3, 14, 15, 19]
Number of T2-weighted lesions	Low	[13, 22]
Black holes (T1-weighted lesions)	More often than in RRMS	[13, 51]
Cortical lesions	Very often	[22–24]
Leptomeningeal enhancement	Often	[37–40]
Dirty appearing white matter	Very often	[13, 57–59]
Slowly expanding/evolving lesions	Often	[63, 67]
Rim-positive lesions	Often	[68–72]
Atrophy	Very often	[77, 78]
MTR from NAWM	Lower than in RRMS	[106, 114]
MTR from NAGM	Lower than in RRMS	[106, 114]
DWI (MD) T2- WI lesions, NAWM, NAGM	Lower than in patients with SPMS	[123, 128]
*H‑MRS (single voxel)*		
Cho/Cr NAWM	Higher compared with RRMS	[133]
NAA/Cr in chronic non-enhancing focal lesions	Lower compared with RRMS	[136]
*CSI*		
Level of glutathione	Lower than in patients with RRMS	[137]
SWI in the thalamus	Lower than in patients with RRMS	[140]
**Spinal cord**		
Number of Gd+ lesions	Very low	[14]
T2-weighted lesions (size, location)	As in RRMS patients (usual)	[89–91]
Diffuse mild hyperintensity	Very often	[89–91]
Atrophy	Very often	[95, 96]
MTR from cervical NAWM and NAGM	Higher than in patients with SPMS	[118]
DWI (FA)	Lower than in healthy control	[129]
DWI (MD)	Higher than in healthy control	[129]

## Conclusion

Primary progressive MS is a less common and distinct form of MS. Differences are observed not only in the pathology and clinical presentation but also in conventional and nonconventional MRI. An awareness of the characteristic MRI features in PPMS patients has relevant clinical applications and may support and simplify the diagnosis of the disease and treatment monitoring.
